# The Gut Microbiome, Inflammation, and Salt-Sensitive Hypertension

**DOI:** 10.1007/s11906-020-01091-9

**Published:** 2020-09-03

**Authors:** Fernando Elijovich, Cheryl L. Laffer, Melis Sahinoz, Ashley Pitzer, Jane F. Ferguson, Annet Kirabo

**Affiliations:** 1 Department of Medicine, Division of Clinical Pharmacology, Vanderbilt University Medical Center, Room 536 Robinson Research Building, Nashville, TN 37232-6602 USA; 2 Department of Medicine, Division of Nephrology, Vanderbilt University Medical Center, Nashville, TN USA; 3 Division of Cardiovascular Medicine, Department of Medicine, Vanderbilt Translational and Clinical Cardiovascular Research Center (VTRACC), Vanderbilt University Medical Center, Nashville, TN USA; 4 Department of Molecular Physiology and Biophysics, Vanderbilt University School of Medicine, Nashville, TN USA

**Keywords:** Salt-sensitive hypertension, Microbiome, Immune activation

## Abstract

**Purpose of Review:**

Salt sensitivity of blood pressure (SSBP) is an independent predictor of death due to cardiovascular events and affects nearly 50% of the hypertensive and 25% of the normotensive population. Strong evidence indicates that reducing sodium (Na^+^) intake decreases blood pressure (BP) and cardiovascular events. The precise mechanisms of how dietary Na^+^ contributes to elevation and cardiovascular disease remain unclear. The goal of this review is to discuss mechanisms of salt-induced cardiovascular disease and how the microbiome may play a role.

**Recent Findings:**

The innate and adaptive immune systems are involved in the genesis of salt-induced hypertension. Mice fed a high-salt diet exhibit increased inflammation with a marked increase in dendritic cell (DC) production of interleukin (IL)-6 and formation of isolevuglandins (IsoLG)-protein adducts, which drive interferon-gamma (IFN-γ) and IL-17A production by T cells. While prior studies have mainly focused on the brain, kidney, and vasculature as playing a role in salt-induced hypertension, the gut is the first and largest location for Na^+^ absorption. Research from our group and others strongly suggests that the gut microbiome contributes to salt-induced inflammation and hypertension.

**Summary:**

Recent studies suggest that alterations in the gut microbiome contribute to salt-induced hypertension. However, the contribution of the microbiome to SSBP and its underlying mechanisms are not known. Targeting the microbiota and the associated immune cell activation could conceivably provide the much-needed therapy for SSBP.

## Introduction

Hypertension is a growing health care burden and is the leading cause of mortality due to myocardial infarction, stroke, heart failure, and chronic kidney disease. Recently, the American Heart Association (AHA) and the American College of Cardiology (ACC) developed new classification criteria that put nearly half of the American US population in the hypertensive category [1]. Accordingly, the prevalence of hypertension is expected to triple among men and double among women. In the USA alone, hypertension accounts for $46 billion in annual health care costs and this is likely to increase. Despite the importance of extensive research on hypertension, its pathogenesis remains elusive and blood pressure control in the general population remains suboptimal [2].

Excess dietary salt is a major risk factor for hypertension and cardiovascular disease [3, 4]. The AHA recommends a maximum of 2300 mg of Na^+^ intake per day; however, less than 10% of the U.S. population observes this recommendation [5, 6]. A meta-analysis by He et al. estimated that modest reductions in Na^+^ intake lower blood pressure and reduce the annual new cases of coronary heart disease and stroke in the USA by 20% [7]. A major problem with excess salt consumption is the presence of salt sensitivity of blood pressure (SSBP) in large numbers of normotensive and hypertensive subjects. SSBP is an independent risk factor for death due to cardiovascular disease. Despite its importance, the pathogenesis of salt sensitivity of blood pressure remains poorly understood. Salt-sensing mechanisms in hypertension involving the kidney, vasculature, and central nervous system have been well studied; however, Na^+^ sensing in the gut and the mechanisms by which commensal microbiota contribute to SSBP have not been well explored. Recent studies including our own have found that the gut microbiome and immune cells can sense Na^+^ and contribute to inflammation and hypertension [8–10].

The intestinal mucosa is the first and main absorption site for excess salt. Dendritic cells (DCs) play a key role in regulating intestinal immune homeostasis in part by surveying the gut epithelial surface for pathogens. DCs survey the mucosa and regulate intestinal immune homeostasis by (1) inducing tolerance to harmless antigens and (2) initiating protective immunity against intestinal pathogens. In this review, we discuss the mechanisms by which these and other immune cells contribute to SSBP and highlight recent studies implicating the gut microbiome in the pathogenesis of the salt-induced cardiovascular disease.

## Salt Sensitivity of Blood Pressure

SSBP is a phenotype observed in some members of mammal species, including humans, characterized by changes in BP that parallel changes in salt intake. It has been possible to create pure homogenous SS and salt-resistant (SR) strains of rodents by inbreeding over many generations, supporting a genetic basis for the SSBP phenotype. As opposed to dichotomization in these rodents, the trait is normally distributed in human populations. Hence, arbitrary cutoffs in changes of BP in response to salt or salt depletion are required for the definition of human SS vs SR. With current dietary or acute salt-loading protocols and accepted cutoffs, the prevalence of SSBP is about 25% in normotensive subjects, 50% in essential hypertension, and as high as 75–80% in hypertensive African Americans.

There are no differences between SS and SR subjects in the handling of a salt load or in plasma volume; both subgroups excrete a salt load equally. The difference between groups is that their equal salt excretion is associated with increased BP in SS but not in SR. Variability in the BP response to salt in humans may be due to the trait being polygenic, to interactions between genetic and environmental factors or to both. Environmental factors known to increase SSBP include low birth weight, aging, obesity, and insulin resistance. The initial impetus for research on the mechanisms of SSBP was to understand a major issue in the regulation of (sodium) Na^+^ metabolism and BP. However, this research became more important once long-term studies uncovered the fact that SSBP was an independent cardiovascular risk factor in normotensive and hypertensive humans, perhaps as potent as hypertension itself [11••, 12••]. In the absence of a unifying mechanism or cause, SSBP remains a risk factor orphan of treatment.

Most early research was an outgrowth of Guyton’s modeling of cardiovascular regulation by salt. His group first proposed the concept of the kidney’s role in long-term BP control by observing that hypertension can develop when the pressure natriuretic balance is impaired. Within this framework of an “infinite gain” for the renal function curve, the model posited that salt would not be able to raise BP unless there was a defect in the regulation of natriuresis. This could theoretically be due to alterations in cell membrane Na^+^ transporters located in the proximal tubule (Na^+^–H^+^ exchanger), distal tubule (Na^+^–Cl^−^ cotransporter), thick ascending limb (Na^+^–K^+^–2Cl^−^ cotransporter), and collecting duct (epithelial Na^+^ channel or ENaC). Any genetic or nongenetic factors affecting the function of these transporters would be associated with fluctuations in systemic BP, Na^+^ retention, and urinary Na^+^ loss. Impairment in renal sodium handling and plasma volume expansion would therefore be major factors in the pathogenesis of salt-sensitive hypertension. However, salt retention or plasma volume expansion is not enhanced in SS animals or humans compared with SR. This is an indispensable intermediate step to support the view that the vasoconstriction of essential hypertension is the consequence of total body autoregulation of blood flow. Instead, the hemodynamic abnormality of SS subjects is an inability to reduce vascular resistance in response to a salt load, whereas such vasodilation in SR is the physiological response that maintains BP unmodified despite the salt-induced increase in cardiac output. The latter observations would support a primary vascular, not renal mechanism.

Research in humans and in strains of rats inbred for SSBP described innumerable abnormalities in most natriuretic/antinatriuretic and vasoconstrictor/vasodilator systems, including but not limited to the renin-angiotensin-aldosterone and sympathetic nervous systems, insulin sensitivity, the endothelins, nitric oxide, reactive oxygen species, dopamine, natriuretic peptides, and CYP450 metabolites of arachidonic acid. More recently, with the advent of techniques for genetic manipulation (congenic and consomic rat strains, zinc finger nuclease-mediated gene-deletion in rats, and transgenic and knockout mice), abnormalities in almost 100 genes have been linked to SSBP [13••]. These genes belong to all the systems mentioned above and to additional ones (prostaglandins, kinins, angiogenesis substances, renal transporters, and their regulatory molecules and the innate and adaptive immune systems). Interestingly, some of these genes can only affect renal function (e.g., uromodulin) and others have been studied by genetically modifying their expression exclusively in vascular smooth muscle (e.g., mineralocorticoid receptor), suggesting the existence of both renal and vascular primary mechanisms in the causation of the SSBP phenotype.

Most recently, knowledge has expanded in terms of the compartments in which Na^+^ is distributed in the body. In addition to the traditional intravascular, interstitial, and intracellular compartments in which Na^+^ is distributed in isoosmolar fashion, it has become evident that there is an interstitial component of Na^+^ storage, which is hyperosmolar, mostly studied in the skin and muscle but probably existing in other tissues and organs as well. There has been the suggestion that regulation of this compartment may be different in SS and SR subjects [14] and this may be important because hyperosmolar Na^+^ may be a key player in the activation of cells of the immune system, as discussed below.

## Salt and Inflammation

For more than 50 years, scientists have convincingly demonstrated that inflammation contributes to hypertension [15, 16]. It is now well established that high dietary Na^+^ polarizes immune cells towards an inflammatory phenotype, enhancing interleukin (IL)-17 production and hypertension [17–19]. T cells infiltrate the kidneys and perivascular space in response to hypertensive stimuli and release inflammatory cytokines that promote renal and vascular dysfunction leading to elevated blood pressure [20–22]. We found that isolevuglandins (IsoLGs) accumulate in murine DCs and human monocytes and modify proteins which act as neoantigens, promoting T cell activation and hypertension [15]. IsoLGs are highly reactive products of lipid oxidation and form covalent bonds to lysine residues leading to post-translational protein modifications [23]. We recently demonstrated that elevated Na^+^ is a potent hypertensive stimulus for IsoLG-adduct formation in DCs in murine models [8]. Na^+^ enters DCs through amiloride-sensitive transporters and is exchanged for calcium (Ca^2+^) via the Na^+^/Ca^2+^ exchanger. Ca^2+^ entry activates protein kinase C (PKC) which in turn phosphorylates the NADPH oxidase subunit p47^*phox*^ [8]. This leads to activation of the NADPH oxidase, increased superoxide (O_2_^·−^), derivative reactive oxygen species (ROS) production, and IsoLG formation [8]. These studies support the premise that elevated Na^+^ increases immune cell activation and that therapeutic strategies to reduce tissue Na^+^ may reduce inflammation and hypertension.

## The Gut Microbiome and Hypertension

Recent evidence shows that gut microbiota play a role in the development of the cardiovascular disease, including hypertension [24, 25]. Germ-free mice are resistant to hypertension and vascular dysfunction and have less renal and vessel infiltration of immune cells after angiotensin II infusion [26]. Transplant of the gut microbiome from hypertensive subjects increases blood pressure in germ-free recipient mice, suggesting a causal role of the gut microbiome in the development of hypertension [27]. The gut microbiome of both hypertensive rats and humans is characterized by an increase in the Firmicutes/Bacteroidetes ratio [28]. This ratio also increases in conditions of obesity and metabolic syndrome in both experimental animals and in human subjects [29, 30]. Importantly, we found that a high-salt diet increased gut colonization by bacteria of the phylum Firmicutes with a resultant increase in the Firmicutes/Bacteroidetes ratio in both humans and mice [9]. These data support a link between dietary sodium, modulation of microbiota, and development of hypertension.

## Salt Sensitivity of Blood Pressure, the Gut Microbiome, and Inflammation

**Fig. 1:**
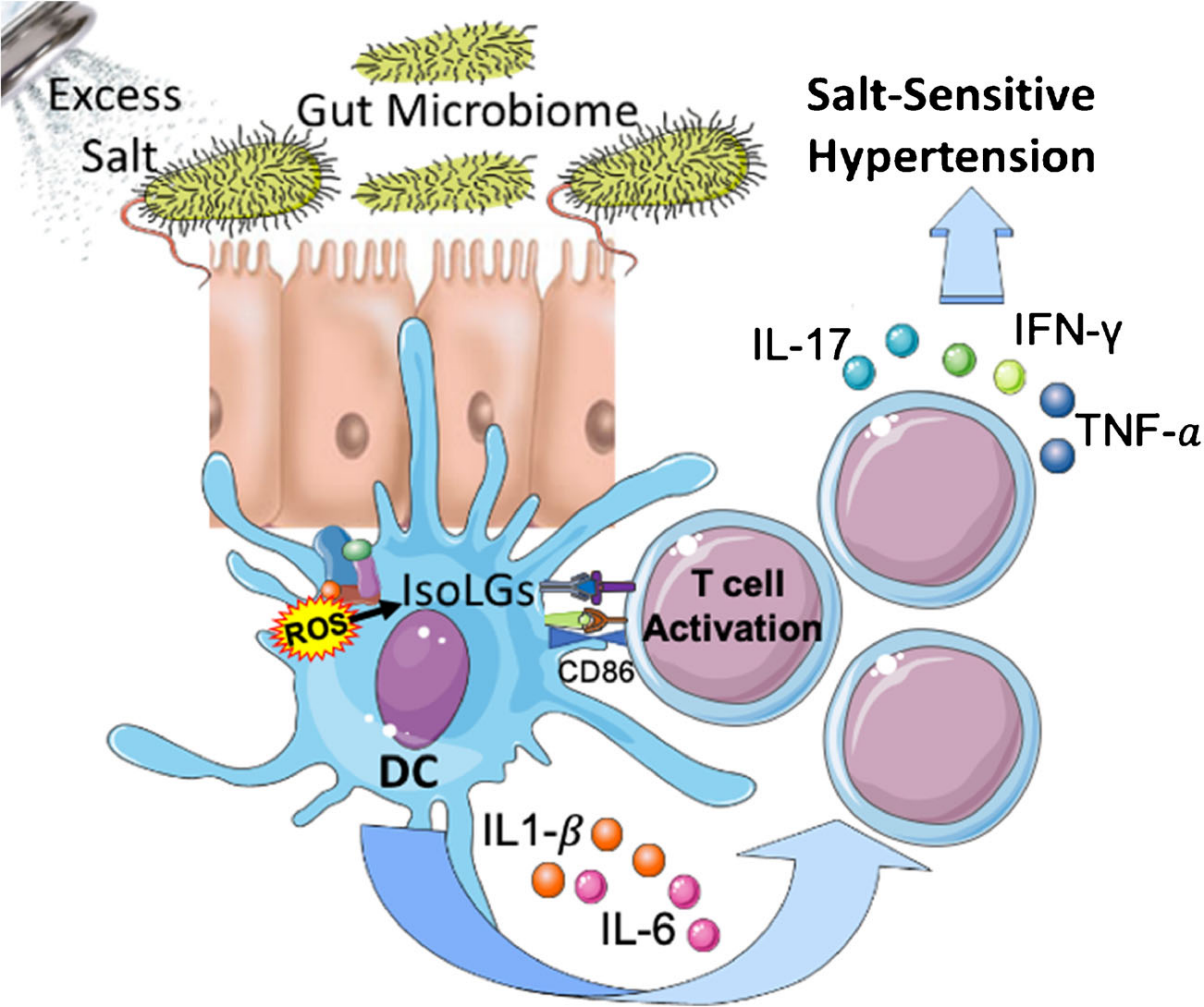
Hypothesized model of how the gut microbiome may contribute to inflammation and salt-sensitive hypertension. Excess dietary salt alters the gut microbiome and activates DCs to produce ROS via NADPH oxidase. ROS production leads to IsoLG-adducted protein formation, presentation of co-stimulatory factor CD86, and secretion of pro-inflammatory factors IL-6 and IL-1*β*. The activated DCs promote T cell activation and stimulate the release of IL-17, TNF-α𝑎, and IFN-γ, leading to hypertension

The intestinal mucosa is the first and main absorption site for excess salt and is richly endowed with immune cells, which sample ingested antigens and induce tolerance and protective immunity against intestinal pathogens. Inbred SS and SR Dahl rats have significant differences in their microbiota composition [31••]. The interaction between their blood pressure phenotype and their microbiomes is complex. Hence, transplantation of SS or SR microbiota to antibiotic-treated SR rats does not affect their blood pressure, whereas, in antibiotic-treated SS rats, transplantation of SR microbiota exaggerates salt-induced hypertension, as if the native SS microbiota exerted a protective effect. In mice, salt depletes *Lactobacillus* spp., and this exerts unquestionable pro-inflammatory effects because therapeutic repletion of these bacteria markedly attenuates either experimental autoimmune encephalitis or salt-sensitive hypertension by modulating T_H_17 cells. Interestingly, a high salt challenge in humans also reduced the intestinal survival of *Lactobacillus* spp., increased TH17 cells, and increased blood pressure [32]. We also have shown that in humans and mice, high salt intake is associated with changes in the gut microbiome. These changes were associated with higher blood pressure in humans and sensitized mice to the effect of subpressor doses of angiotensin II [9]. Furthermore, mice fed a high-salt diet exhibit increased intestinal and vascular inflammation with a marked increase in the B7 ligand CD86 and formation of IsoLG-protein adducts in DCs, which drive interferon-gamma (IFN-γ) and IL-17A production by T cells (Fig. 1)

. Adoptive transfer of fecal material from conventionally housed high salt-fed mice to germ-free mice predisposed them to increased inflammation and hypertension [9]. The aforementioned studies in humans have been conducted in a small number of subjects. Therefore, results must be replicated in a large diverse sample of men and women to firmly establish the effects of Na^+^ on the microbiome and on adaptive immunity. Following this, the mechanisms by which microbiome-induced inflammation in the gut is translated into salt sensitivity of blood pressure, a phenotype thought to reflect renal or vascular abnormalities, will have to be established. It is conceivable that differential microbiome responses to salt account for the variability of salt sensitivity of blood pressure in human populations.

## Summary and Future Perspectives

Recent evidence points to the role of the gut microbiome in salt-induced inflammation and hypertension. More research is warranted to investigate the exact mechanisms by which excess salt intake causes alterations in microbiota and promotes innate and adaptive immune system activation which then leads to salt-sensitive hypertension. The differences in the gut microbiome response to salt among individuals also need exploration. Therapeutic interventions targeting the microbiota and the associated activation in innate and adaptive immunity could serve as potential therapies for SSBP in the future.
